# Supplementary material for: MIL-53 Metal–Organic Framework as a Flexible Cathode for Lithium-Oxygen Batteries
Source: Materials (Basel). 2021 Aug 17;14(16):4618. doi: 10.3390/ma14164618 (PMC8399480; doi:10.3390/ma14164618)
Supplement: Supplementary file 1 [file materials-14-04618-s001.zip › materials-1318416-supplementary.pdf]

materials

MDPI

Supplementary materials

MIL-53 Metal–Organic Framework as a flexible cathode for lithium-oxygen batteries

Yujie Zhang [1], Ben Gikonyo [1,2,3], Hicham Khodja [1], Magali Gauthier [1], Eddy Foy [1], Bernard Goetz [3], Christian Serre [3], Servane Coste-Leconte [4], Vanessa Pimenta [3] and Suzy Surblé [1,*]

1 Université Paris-Saclay, CEA, CNRS, NIMBE, 91191 Gif-sur-Yvette, France; yujie.zhang@cea.fr (Y.Z.); ben.gikonyo@univ-lyon1.fr (B.G.); hicham.khodja@cea.fr (H.K.); magali.gauthier@cea.fr (M.G.); eddy.foy@cea.fr (E.F.)
2 Laboratoire des Multimatériaux et Interfaces, Université Lyon, Université Claude Bernard Lyon 1, UMR CNRS 5615, 69622 Villeurbanne, France
3 Institut des Matériaux Poreux de Paris (IMAP), ESPCI Paris, Ecole Normale Supérieure de Paris, CNRS, PSL University, 75005 Paris, France; vanessa.pereira-pimenta@espci.fr (V.P.); bernard.goetz@espci.fr (B.G.); christian.serre@espci.fr (C.S.)
4 Institut National des Sciences et Techniques Nucléaires, Unité d'Enseignement de Saclay, CEA, 91191 Gif-sur-Yvette, France; servane.coste-leconte@cea.fr
* Correspondence: suzy.surble@cea.fr; Tel.: +33-01-6908-8190

1- *XRD patterns of MIL-53*

The XRD patterns were collected under air atmosphere and after heating the compounds. For the as-synthesized form (MIL-53-as), we observe an additional peak near 17.5° corresponding to the disordered free acid enclosed into the pore. MIL-53-lp cannot be isolated in this condition, since water molecules are adsorbed from atmosphere. Therefore, in both cases, we observe the coexistence of both the MIL-53-np and MIL-53-lp forms. When the solid is fully hydrated, only the MIL-53-np is identified.

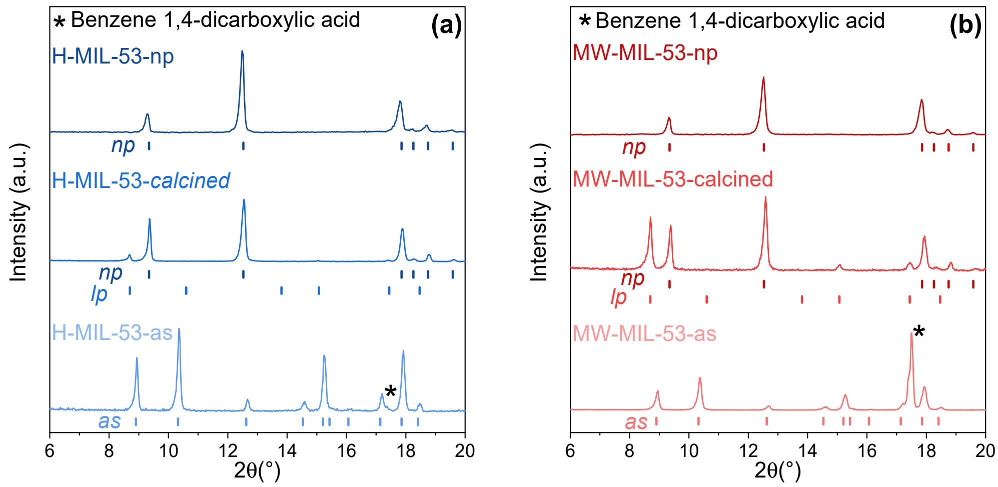

Figure S1. Powder patterns (Cu-Kα) of as-synthesized (as), calcined at 360°C (or lp) and fully hydrated (or np) forms of (**a**) H-MIL-53 and (**b**) MW-MIL-53. Bragg peaks of MIL-53-as, MIL-53-lp and MIL-53-np forms are indicated by tic marks (light to dark colors).

2- *TGA analysis*

The thermogravimetric curves for both as-synthesized and dried forms of the MIL-53 show a similar trend, in agreement with the literature [22]. The departure of the free disordered terephthalic acid molecules occurs in three steps within the range 250 – 450 °C for both as-synthetized materials. The formula calculated from TGA measurements is $Al(OH)(O_2C\text{-}C_4H_4\text{-}CO_2)\cdot x(HO_2C\text{-}C_4H_4\text{-}CO_2H)$, where x = 0.7 and where 1.3 BDC molecules are encapsulated within the pores of H-MIL-53 and MW-MIL-53 respectively (obs. 63.4 % / calc. 64.1 % and obs. 49.3 % / calc. 49.1 % for hydrothermal or microwave syntheses respectively). Around 560 °C, the elimination of the BDC linkers from the framework leads to the formation of Al_2O_3 (obs. 48.5 % / calc. 47.9 % for –H-MIL-53 and obs. 35.01 % / calc. 37.22 % for MW-MIL-53).

The thermal behavior of hydrated forms (*np*) is characterized by two weight losses. The first loss corresponds to the dehydration stage. For both syntheses, we observe around 0.7 water molecules encapsulated in the pores (weight loss of 6.0% and 5.5% for H-MIL-53 and MW-MIL-53 calc. 5.7%). The second is assigned to the collapse of the framework and the formation of Al_2O_3 (obs. 70.9% / calc. 70.6 % for H-MIL-53 and obs. 71.3% / calc. 71.6 % for MW-MIL-53).

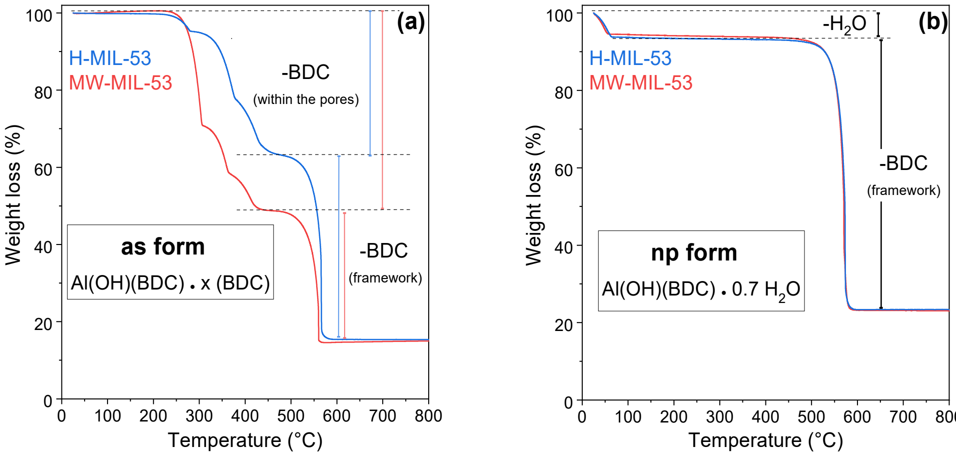

Figure S2. TGA analysis of terephthalate solids (a) as-synthesized and (b) after activation of aluminum obtained through the hydrothermal route (H-MIL-53, blue) and microwave method (MW-MIL-53, red).

2 - *Breathing transitions studies*

We first studied the breathing transition of the MOFs materials when solvent or guest molecules are present in the MIL-53 electrode (NMP and DME solvents, PVDF binder and LiTFSI electrolyte). Figure S3a shows the XRD patterns obtained when the MIL-53 solid is in contact respectively with an excess of NMP and DME solvents, the binder (10 wt. % PVDF@ NMP) and the electrolyte (LiTFSI@DME). As soon as the powder is in contact with an excess of solvent, a breathing transition occurs. XRD patterns reveal an orthorhombic system for both MIL-53-NMP and MIL-53-DME. When the binder is added, additional peaks are observed compared to the pattern of MIL-53-NMP. These latter peaks disappear by drying the compounds at 80°C under vacuum (temperature corresponding to the MIL-53 electrode's preparation). Only the fingerprint of the MIL-53-PVDF phase appears after calcination. The solids in contact with PVDF and LiTFSI crystallize in a monoclinic cell. The cell parameters are given in Table S1.

Table S1. Cell parameters obtained by pattern matching refinement for H-MIL-53 wetted with several solvents or salt/binder dissolved in solvents.

	lp form[1]	np form[1]	NMP	DME	PVDF@NMP dried	LiTFSI@DME dried
System	orthorhombic	monoclinic	orthorhombic	orthorhombic	monoclinic	monoclinic
Space group	Imma (n°74)	Cc (n°9)	mma (n°74)	Imma (n°74)	Cc (n°9)	Cc (n°9)
a / Å	16.675(3)	19.513(2)	15.96(1) Å	19.06(1) Å	18.31(1)	21.66(1)
b / Å	12.813(2)	7.612(1)	13.86(1) Å	9.26(1) Å	10.08(1)	8.08(1)
c / Å	6.608(1)	6.576(1)	6.63(1) Å	6.53(1) Å	10.08(1)	9.65(1)
β / °	-	104.24(1)	-	-	106.31(2)	103.81(4)
V / Å^3	1411.9(4)	946.7(2)	1468.9(3)	1153.9(9)	1551.4(3)	1642.1(2)

[1] parameter cells reported in the literature [1]

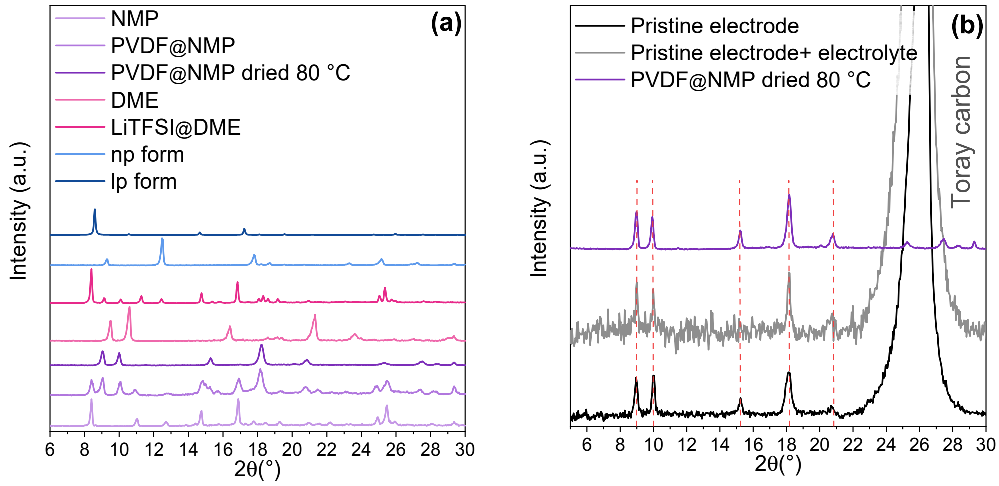

Figure S3. (a) Experimental X-ray powder diffraction patterns (Kα-Cu) for H-MIL-53 exposed respectively to NMP and DME solvents, impregnated with the binder (PVDF dissolved in NMP) before and after drying at 80°C and wetted with the electrolyte LiTFSI in DME. For comparison, the XRD diagrams of MIL-53-np and MIL-53-lp were added. **(b)** Comparison of XRD patterns of pristine electrodes (without or with electrolyte) with the MIL-53 powder impregnated with the binder and dried.

3 - Galvanostatic Li-O_2 batteries with MIL-53 and Super P based electrodes

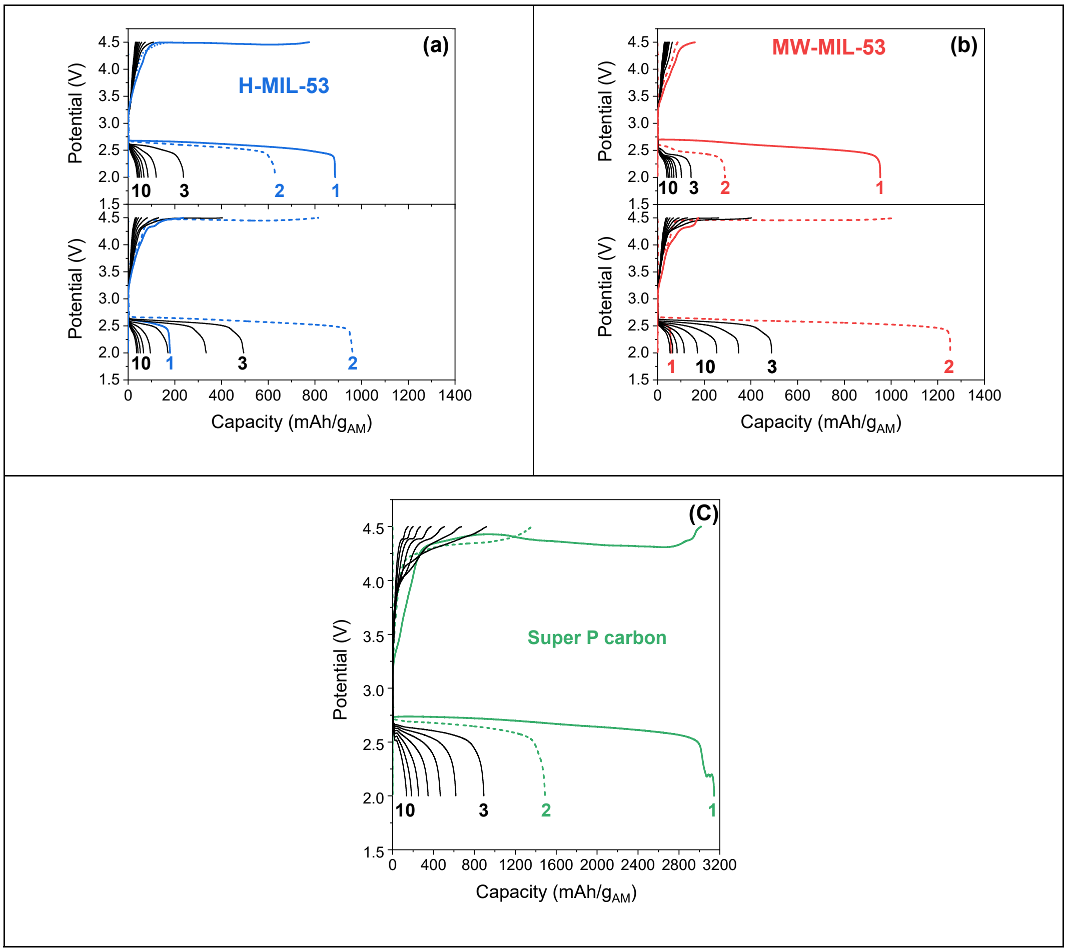

Figure S4. Galvanostatic Li-O_2 discharge-charge cycles at 50 mAh/g_{AM} with (**a**) MW-MIL-53, (**b**) H-MIL-53 and (**c**) Super P carbon electrodes with a constant decrease of capacity over cycling or with the second capacity higher than the first capacity. The charge-discharge profiles of cycle 1 and 2 are represented with full solid lines and dash lines respectively, while the profiles from cycle 3 to 10 are represented with black solid line for all electrodes.

4 – Ex situ characterizations

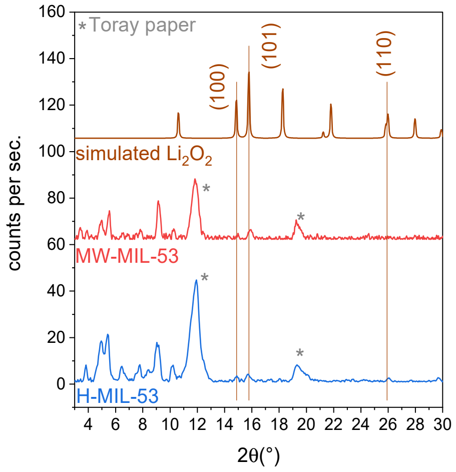

Figure S5. *Ex situ* XRD patterns (Mo-Kα) of H-MIL-53 (blue) and MW-MIL-53 (red) electrodes after 1 discharge.

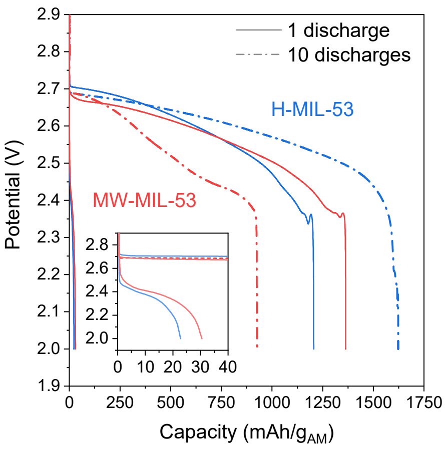

Figure S6. Discharge profiles of Li-O_2 cell with H-MIL-53 (blue) and MW-MIL-53 (red) electrodes after 1 (dash dot line) and 10 discharges (solid line). To compare the first capacities, the first discharge profiles for were added for both electrodes.
